# Supplementary material for: The Effects of Time-Restricted Eating on Fat Loss in Adults with Overweight and Obese Depend upon the Eating Window and Intervention Strategies: A Systematic Review and Meta-Analysis
Source: Nutrients. 2024 Oct 5;16(19):3390. doi: 10.3390/nu16193390 (PMC11478505; doi:10.3390/nu16193390)
Supplement: Supplementary file 1 [file nutrients-16-03390-s001.zip › Supplement S1.pdf]

The effects of time-restricted eating on fat loss in adults with overweight and obese depend upon the eating window and intervention strategies: A systematic review and meta-analysis Yixun Xie

TABLE S1 Subgroup analysis results regarding the effects of TRE on all results.

Outcomes	Variables	No. of studies	WMD (95%CI)	*P*-value	Test of heterogeneity		
					χ^2	*P*-value	I^2(%)
Fat mass, kg	**Eating window**						
	6 to 8 hours	13	-1.66(-2.31, -1.01)	0.000	25.91	0.011	53.70%
	10 to 12 hours	5	-0.65(-1.39, -0.90)	0.085	0.84	0.933	0.00%
	**Study durations**						
	≤ 39 weeks	15	-1.36(-2.00, -0.72)	0.000	31.99	0.004	56.20%
	≥ 39 weeks	3	-1.51(-2.52, -0.51)	0.003	1.61	0.448	0.00%
	**Age**						
	young adults	13	-1.61(-2.23, -0.99)	0.000	22.5	0.032	46.70%
	middle-aged adults	3	-0.64(-1.27, -0.01)	0.045	0.25	0.883	0.00%
	older adults	2	-1.80(-4.66, 1.06)	0.218	0.18	0.668	0.00%
	**Intervention strategies**						
	TRE-only	8	-1.26(-1.89, -0.62)	0.000	9.53	0.217	26.50%
	TRE Combined with CR	4	-0.83(-1.53, -0.12)	0.022	1.68	0.642	0.00%
	TRE Combined exercise	3	-3.07(-6.02, -0.12)	0.041	1.28	0.526	0.00%
	TRE Combined with CR and exercise recommended	3	-1.87(-3.17, -0.57)	0.005	6.03	0.049	66.80%
Lean mass, kg	**Eating window**						
	6 to 8 hours	13	-0.74(-0.95, -0.53)	0.000	10.05	0.612	0.00%
	10 to 12 hours	2	-0.01(-0.60, 0.58)	0.968	0.82	0.366	0.00%
	**Study durations**						
	≤ 39 weeks	12	-0.73(-0.95, -0.51)	0.000	10.37	0.498	0.00%
	≥ 39 weeks	3	-0.25(-0.84, 0.35)	0.416	2.65	0.266	24.60%
	**Age**						
	young adults	10	-0.53(-0.77, -0.28)	0.000	9.52	0.391	5.40%
	middle-aged adults	3	-1.09(-1.51, -0.66)	0.000	0.81	0.667	0.00%
	older adults	2	0.10(-2.45, 2.66)	0.936	0.4	0.529	0.00%
	**Intervention strategies**						
	TRE-only	7	-1.04(-1.36, -0.72)	0.000	2.29	0.891	0.00%
	TRE Combined with CR	3	-0.30(-0.85, 0.26)	0.293	0.29	0.865	0.00%
	TRE Combined exercise	2	-0.01(-1.78, 1.76)	0.989	0.14	0.712	0.00%
	TRE Combined with CR and exercise recommended	3	-0.30(-0.82, 0.22)	0.261	3.82	0.148	47.70%
Body mass, kg	**Eating window**						
	6 to 8 hours	13	-2.73(-3.42, 2.05)	0.000	19.69	0.073	39.10%
	10 to 12 hours	6	-0.67(-1.44, 0.11)	0.091	0.45	0.994	0.00%
	**Study durations**						
	≤ 39 weeks	15	-2.13(-3.01, -1.25)	0.000	41.4	0.000	66.20%
	≥ 39 weeks	4	-2.02(-3.94, -0.09)	0.007	5.4	0.145	44.40%
	**Age**						
	young adults	18	-2.24(-3.13, -1.36)	0.000	41.43	0.000	68.60%
	middle-aged adults	2	-1.13(-2.31, 0.04)	0.058	0.01	0.933	0.00%
	older adults	3	-1.79(-4.87, 1.28)	0.252	0.01	0.997	0.00%
	**Intervention strategies**						
	TRE-only	8	-2.39(-3.42, -1.35)	0.000	9.84	0.198	28.90%
	TRE Combined with CR	5	-1.22(-2.13, -0.30)	0.009	5.14	0.273	22.20%

	TRE Combined exercise	3	-3.76(-7.52, 0.01)	0.051	0.99	0.609	0.00%
	TRE Combined with CR and exercise recommended	3	-2.48(-4.15, -0.81)	0.004	8.45	0.015	76.30%
BMI	**Eating window**						
	6 to 8 hours	9	-0.98(-1.44, -0.52)	0.000	23.04	0.003	65.30%
	10 to 12 hours	3	-0.27(-0.58, 0.05)	0.097	0.08	0.963	0.00%
	**Study durations**						
	≤ 39 weeks	9	-0.70(-1.13, -0.28)	0.001	28.1	0.000	71.50%
	≥ 39 weeks	3	-0.98(-1.85, -0.11)	0.027	3.29	0.193	39.20%
	**Age**						
	young adults	8	-0.86(-1.37, -0.34)	0.000	26.54	0.000	73.60%
	middle-aged adults	1	-0.34(-0.68, 0.00)	0.051	0	.	.%
	older adults	3	-0.82(-1.54, -0.11)	0.025	0.75	0.689	0.00%
	**Intervention strategies**						
	TRE-only	6	-0.89(-1.36, -0.42)	0.000	14.65	0.012	65.90%
	TRE Combined with CR	4	-0.31(-0.60, 0.57)	0.037	0.65	0.884	0.00%
	TRE Combined exercise	2	-2.47(-5.51, 0.57)	0.111	3.68	0.055	72.80%
Total cholesterol, mg dL	**Eating window**						
	6 to 8 hours	8	-0.99(-6.87, 4.90)	0.924	12.89	0.075	45.70%
	10 to 12 hours	3	4.79(-0.08, 9.66)	0.054	0.82	0.664	0.00%
	**Study durations**						
	≤ 39 weeks	9	1.26(-4.25, 6.77）	0.654	15.85	0.045	49.50%
	≥ 39 weeks	2	0.59(-6.27, 7.44)	0.866	0.19	0.667	0.00%
	**Age**						
	young adults	10	1.63(-2.89, 6.16)	0.479	15.3	0.083	41.20%
	middle-aged adults	1	-4.53(-18.05, 8.99)	0.511	0	.	.%
	**Intervention strategies**						
	TRE-only	4	3.02(-1.55, 7.59)	0.286	2.98	0.394	0.00%
	TRE Combined with CR	3	2.31(-5.56, 10.17)	0.279	2.04	0.361	1.90%
	TRE Combined exercise	3	-2.90(-18.46, 12.65)	0.714	9.86	0.007	79.70%
	TRE Combined with CR and exercise recommended	1	6.00(-10.50, 22.50)	0.476	0	.	.%
HDL, mg dL	**Eating window**						
	6 to 8 hours	9	0.69(-0.48, 1.85)	0.248	8.44	0.391	5.30%
	10 to 12 hours	3	0.37(-2.49, 3.24)	0.798	5.48	0.065	63.50%
	**Study durations**						
	≤ 39 weeks	10	0.31(-1.05, 1.67)	0.654	13.11	0.158	31.30%
	≥ 39 weeks	2	1.58(-0.57, 3.74)	0.150	0.02	0.881	0.00%
	**Age**						
	young adults	11	0.91(-0.45, 2.26)	0.103	12.15	0.145	34.20%
	middle-aged adults	3	-0.81(-3.07, 1.44)	0.353	0.77	0.680	0.00%
	**Intervention strategies**						
	TRE-only	6	0.13(-1.02, 1.28)	0.824	2.04	0.844	0.00%
	TRE Combined with CR	2	2.38(0.17, 4.60)	0.035	0.87	0.352	0.00%
	TRE Combined exercise	3	1.59(-5.18, 8.36)	0.645	7.94	0.019	74.80%
	TRE Combined with CR and exercise recommended	1	0.00(-4.35, 4.35)	1.000	0	.	.%
Triglycerides, mg dL	**Eating window**						
	6 to 8 hours	8	-4.68(-9.01, -0.36)	0.034	6.22	0.514	0.00%
	10 to 12 hours	3	1.28(-1.67, 4.23)	0.396	1.09	0.580	0.00%

The effects of time-restricted eating on fat loss in adults with overweight and obese depend upon the eating window and intervention strategies: A systematic review and meta-analysis Yixun Xie

**Study durations**						
≤ 39 weeks	9	-1.30(-5.05, 2.45)	0.497	11.93	0.154	32.90%
≥ 39 weeks	2	-0.84(-16.57, 14.89)	0.916	0.36	0.551	0.00%
**Age**						
young adults	8	-0.17(-2.70, 2.36)	0.895	6.04	0.535	0.00%
middle-aged adults	3	-12.57(-30.90, 5.76)	0.179	4.61	0.100	56.60%
**Intervention strategies**						
TRE-only	6	-1.85(-7.83, 4.14)	0.545	6.49	0.262	22.90%
TRE Combined with CR	2	-2.52(-16.42, 11.38)	0.695	1.06	0.304	5.20%
TRE Combined exercise	2	3.10(-12.35, 18.54)	0.677	4.29	0.038	76.70%
TRE Combined with CR and exercise recommended	1	-6.00(-30.76, 18.76)	0.635	0	.	.%

Note: WMD, weighted mean difference TRE, rime restricted eating; CR, calorie restriction.

The effects of time-restricted eating on fat loss in adults with overweight and obese depend upon the eating window and intervention strategies: A systematic review and meta-analysis Yixun Xie

Author(s):
Question: TRE compared to CON for Obesity
Setting:
Bibliography: . TRE versus CON for Obesity. Cochrane Database of Systematic Reviews [Year], Issue [Issue].

Certainty assessment							№ of patients		Effect		Certainty	Importance
№ of studies	Study design	Risk of bias	Inconsistency	Indirectness	Imprecision	Other considerations	TRE	CON	Relative (95% CI)	Absolute (95% CI)		
**Body mass, kg**												
19	randomised trials	serious[a]	not serious	not serious	not serious	none	624	571	-	MD **2.11 lower** (2.89 lower to 1.33 lower)	⨁⨁⨁◯ Moderate	
**BMI, kg/m²**												
12	randomised trials	serious[b]	not serious	not serious	not serious	none	424	377	-	MD **0.75 lower** (1.12 lower to 0.38 lower)	⨁⨁⨁◯ Moderate	
**Fat mass, kg**												
18	randomised trials	not serious	not serious	not serious	not serious	none	559	509	-	MD **1.4 lower** (1.94 lower to 0.85 lower)	⨁⨁⨁⨁ High	
**Body Fat, %**												
16	randomised trials	not serious	not serious	not serious	not serious	none	534	475	-	MD **0.48 lower** (0.83 lower to 0.13 lower)	⨁⨁⨁⨁ High	
**Lean mass, kg**												
15	randomised trials	not serious	not serious	not serious	not serious	none	465	416	-	MD **0.64 lower** (0.87 lower to 0.4 lower)	⨁⨁⨁⨁ High	
**Waist circumference, cm**												
12	randomised trials	not serious	not serious	not serious	not serious	none	436	398	-	MD **2.14 lower** (2.88 lower to 1.4 lower)	⨁⨁⨁⨁ High	
**Total cholesterol, mg/dL**												
11	randomised trials	not serious	not serious	not serious	not serious	none	384	347	-	MD **1.2 higher** (3.07 lower to 5.47 higher)	⨁⨁⨁⨁ High	
**HDL, mg/dl**												
12	randomised trials	not serious	not serious	not serious	not serious	none	400	363	-	MD **0.53 higher** (0.6 lower to 1.65 higher)	⨁⨁⨁⨁ High	
**LDL, mg/dL**												
11	randomised trials	not serious	not serious	not serious	not serious	none	389	353	-	MD **2.7 higher** (0.17 higher to 5.22 higher)	⨁⨁⨁⨁ High	
**Triglycerides, mg/dL**												
11	randomised trials	not serious	not serious	not serious	not serious	none	389	353	-	MD **1.09 lower** (4.31 lower to 2.13 higher)	⨁⨁⨁⨁ High	

CI: confidence interval; **MD:** mean difference

Explanations

a. 50%<I2<75%
b. 50%<I2<75%

FIGURE S1 Certainty of the evidence
