# Supplementary material for: The Effects of Time-Restricted Eating on Fat Loss in Adults with Overweight and Obese Depend upon the Eating Window and Intervention Strategies: A Systematic Review and Meta-Analysis
Source: Nutrients. 2024 Oct 5;16(19):3390. doi: 10.3390/nu16193390 (PMC11478505; doi:10.3390/nu16193390)
Supplement: Supplementary file 1 [file nutrients-16-03390-s001.zip › Supplement S2.pdf]

The effects of time-restricted eating on fat loss in adults with overweight and obese depend upon the eating window and intervention strategies: A systematic review and meta-analysis Yixun Xie

Search query history for Seven electronic databases:

Cochrane

#1 (Time Restricted Eating):ti,ab,kw OR (Eating, Time Restricted):ti,ab,kw OR (Time Restricted Fasting):ti,ab,kw OR (Fasting, Time Restricted):ti,ab,kw OR (Restricted Fastings, Time):ti,ab,kw OR (Time Restricted Feeding):ti,ab,kw OR (Feeding, Time Restricted):ti,ab,kw OR (Time Restricted Feedings):ti,ab,kw OR (Time restricted meal):ti,ab,kw OR (Time restricted diet):ti,ab,kw OR (Early Time-Restricted Eating):ti,ab,kw 1780

#2 MeSH descriptor: [Obesity] explode all trees 21365

#3 MeSH descriptor: [Overweight] explode all trees 25047

#4 (obesity):ti,ab,kw OR (overweight):ti,ab,kw OR (excess weight):ti,ab,kw OR (obese adults):ti,ab,kw OR (adult with obesity):ti,ab,kw OR (adult obesity):ti,ab,kw OR (obesity in adult):ti,ab,kw OR (obese):ti,ab,kw OR (adiposity):ti,ab,kw 62667

#5 #2 OR #3 OR #4 62768

#6 #5 AND #1 in Trials 705

Embase

#8. #7 AND 'human'/de 441

#7. #5 AND #6 586

#6. #2 OR #4 888,341

#5. #1 OR #3 1,069

#4. 'obesity'/exp OR 'obesity' 842,252

#3. 'time restricted eating'/exp OR 'time restricted eating' 441

#2. 'obesity'/exp OR obesity OR overweight:ab,ti OR 'excess weight':ab,ti OR 'obese adults':ab,ti OR 'adult with obesity':ab,ti OR 'adult obesity':ab,ti OR 'obesity in adult':ab,ti OR obese:ab,ti OR adiposity:ab,ti 888,341

#1. 'time restricted eating':ab,ti OR 'eating, time restricted':ab,ti OR 'time restricted fasting':ab,ti OR 'fasting, time restricted':ab,ti OR 'restricted fastings, time':ab,ti OR 'time restricted feeding':ab,ti OR 'feeding, time restricted':ab,ti OR 'time restricted feedings':ab,ti OR 'time restricted meal':ab,ti OR 'time restricted diet':ab,ti OR 'early time-restricted eating':ab,ti 1,026

Medline

The effects of time-restricted eating on fat loss in adults with overweight and obese depend upon the eating window and intervention strategies: A systematic review and meta-analysis Yixun Xie

S3 (Obesity OR Overweight OR Excess Weight OR Obese Adults OR Adult with Obesity OR Adult Obesity OR Obesity in Adult OR Obese OR Adiposity) AND (S1 AND S2) EBSCOhost Research Databases 202 MEDLINE with Full Text
S2 Obesity OR Overweight OR Excess Weight OR Obese Adults OR Adult with Obesity OR Adult Obesity OR Obesity in Adult OR Obese OR Adiposity- EBSCOhost Research Databases 496,774 MEDLINE with Full Text
S1 Time Restricted Eating OR Eating, Time Restricted OR Time Restricted Fasting OR Fasting, Time Restricted OR Restricted Fastings, Time OR Time Restricted Feeding OR Feeding, Time Restricted OR Time Restricted Feedings OR Time Restricted Meal OR Time Restricted Diet OR Early Time-Restricted Eating EBSCOhost Research Databases 578 MEDLINE with Full Text

PubMed

Search number Query Sort By Filters Search Details Results Time
6 ((("Obesity"[Mesh]) OR ("Overweight"[Mesh])) OR ((((((((Obesity[Title/Abstract]) OR (Overweight[Title/Abstract])) OR (Excess Weight[Title/Abstract])) OR (Obese Adults[Title/Abstract])) OR (Adult with Obesity[Title/Abstract])) OR (Adult Obesity[Title/Abstract])) OR (Obesity in Adult[Title/Abstract])) OR (Obese[Title/Abstract])) OR (Adiposity[Title/Abstract]))) AND ((((((((Time Restricted Fasting[Title/Abstract]) OR (Fasting, Time Restricted[Title/Abstract])) OR (Restricted Fastings, Time[Title/Abstract])) OR (Time Restricted Feeding[Title/Abstract])) OR (Feeding, Time Restricted[Title/Abstract])) OR (Time Restricted Feedings[Title/Abstract])) OR (Time Restricted Meal[Title/Abstract])) OR (Time Restricted Diet[Title/Abstract])) OR (Early Time-Restricted Eating[Title/Abstract])) ("Obesity"[MeSH Terms] OR "Overweight"[MeSH Terms] OR ("Obesity"[Title/Abstract] OR "Overweight"[Title/Abstract] OR "excess weight"[Title/Abstract] OR "obese adults"[Title/Abstract] OR (("Adult"[MeSH Terms] OR "Adult"[All Fields] OR "Adults"[All Fields] OR "adult s"[All Fields]) AND "with obesity"[Title/Abstract]) OR "adult obesity"[Title/Abstract] OR (("obeses"[All Fields] OR "Obesity"[MeSH Terms] OR "Obesity"[All Fields] OR "Obese"[All Fields] OR "obesities"[All Fields] OR "obesity s"[All Fields]) AND "in adult"[Title/Abstract]) OR "Obese"[Title/Abstract] OR "Adiposity"[Title/Abstract])) AND ("time restricted fasting"[Title/Abstract] OR "fasting time restricted"[Title/Abstract] OR ((("restrict"[All Fields] OR "Restricted"[All Fields] OR "restricting"[All Fields] OR "restriction"[All Fields] OR "restrictions"[All Fields] OR "restrictive"[All Fields] OR "restrictiveness"[All Fields] OR "restricts"[All Fields]) AND ("fasted"[All Fields] OR "Fasting"[MeSH Terms] OR "Fasting"[All Fields] OR "fastings"[All Fields] OR "fasts"[All Fields])) AND "Time"[Title/Abstract]) OR "time restricted feeding"[Title/Abstract] OR "feeding time restricted"[Title/Abstract] OR "time restricted feedings"[Title/Abstract] OR "time restricted meal"[Title/Abstract] OR "time restricted diet"[Title/Abstract] OR "early time restricted eating"[Title/Abstract]) 613
5 (("Obesity"[Mesh]) OR ("Overweight"[Mesh])) OR ((((((((Obesity[Title/Abstract]) OR (Overweight[Title/Abstract])) OR (Excess Weight[Title/Abstract])) OR (Obese Adults[Title/Abstract])) OR (Adult with Obesity[Title/Abstract])) OR (Adult Obesity[Title/Abstract])) OR (Obesity in Adult[Title/Abstract])) OR (Obese[Title/Abstract])) OR (Adiposity[Title/Abstract])) "Obesity"[MeSH Terms] OR "Overweight"[MeSH Terms] OR ("Obesity"[Title/Abstract] OR "Overweight"[Title/Abstract] OR "excess weight"[Title/Abstract] OR "obese adults"[Title/Abstract] OR (("Adult"[MeSH Terms] OR "Adult"[All Fields] OR "Adults"[All Fields] OR "adult s"[All Fields]) AND "with obesity"[Title/Abstract]) OR "adult obesity"[Title/Abstract] OR (("obeses"[All Fields] OR "Obesity"[MeSH Terms] OR "Obesity"[All Fields] OR "Obese"[All Fields] OR "obesities"[All Fields]

OR "obesity s"[All Fields]) AND "in adult"[Title/Abstract]) OR "Obese"[Title/Abstract] OR "Adiposity"[Title/Abstract]) 489,917

4 ((((((((Time Restricted Fasting[Title/Abstract]) OR (Fasting, Time Restricted[Title/Abstract])) OR (Restricted Fastings, Time[Title/Abstract])) OR (Time Restricted Feeding[Title/Abstract])) OR (Feeding, Time Restricted[Title/Abstract])) OR (Time Restricted Feedings[Title/Abstract])) OR (Time Restricted Meal[Title/Abstract])) OR (Time Restricted Diet[Title/Abstract])) OR (Early Time-Restricted Eating[Title/Abstract]) "time restricted fasting"[Title/Abstract] OR "fasting time restricted"[Title/Abstract] OR ((("restrict"[All Fields] OR "Restricted"[All Fields] OR "restricting"[All Fields] OR "restriction"[All Fields] OR "restrictions"[All Fields] OR "restrictive"[All Fields] OR "restrictiveness"[All Fields] OR "restricts"[All Fields]) AND ("fasted"[All Fields] OR "Fasting"[MeSH Terms] OR "Fasting"[All Fields] OR "fastings"[All Fields] OR "fasts"[All Fields])) AND "Time"[Title/Abstract]) OR "time restricted feeding"[Title/Abstract] OR "feeding time restricted"[Title/Abstract] OR "time restricted feedings"[Title/Abstract] OR "time restricted meal"[Title/Abstract] OR "time restricted diet"[Title/Abstract] OR "early time restricted eating"[Title/Abstract] 1,781

3 ((((((((Obesity[Title/Abstract]) OR (Overweight[Title/Abstract])) OR (Excess Weight[Title/Abstract])) OR (Obese Adults[Title/Abstract])) OR (Adult with Obesity[Title/Abstract])) OR (Adult Obesity[Title/Abstract])) OR (Obesity in Adult[Title/Abstract])) OR (Obese[Title/Abstract])) OR (Adiposity[Title/Abstract])

"Obesity"[Title/Abstract] OR "Overweight"[Title/Abstract] OR "excess weight"[Title/Abstract] OR "obese adults"[Title/Abstract] OR (("Adult"[MeSH Terms] OR "Adult"[All Fields] OR "Adults"[All Fields] OR "adult s"[All Fields]) AND "with obesity"[Title/Abstract]) OR "adult obesity"[Title/Abstract] OR (("obeses"[All Fields] OR "Obesity"[MeSH Terms] OR "Obesity"[All Fields] OR "Obese"[All Fields] OR "obesities"[All Fields] OR "obesity s"[All Fields]) AND "in adult"[Title/Abstract]) OR "Obese"[Title/Abstract] OR "Adiposity"[Title/Abstract] 441,164

2 "Overweight"[Mesh] "Overweight"[MeSH Terms] 283,317

1 "Obesity"[Mesh] "Obesity"[MeSH Terms] 271,627

Scopus

(TITLE-ABS-KEY ("Time Restricted Eating*" OR "Eating, Time Restricted*" OR "Time Restricted Fasting*" OR "Fasting, Time Restricted*" OR "Restricted Fastings, Time*" OR "Time Restricted Feeding*" OR "Feeding, Time Restricted*" OR "Time Restricted Feedings*" OR "Time Restricted Meal*" OR "Time Restricted Diet*" OR early "Time-Restricted Eating*") AND TITLE-ABS-KEY ("Obesity*" OR "Overweight*" OR "Excess Weight*" OR "Obese Adults*" OR "Adult with Obesity*" OR "Adult Obesity*" OR "Obesity in Adult*" OR "Obese*" OR "Adiposity*")) 227

Sportdisus

S3 (Obesity OR Overweight OR Excess Weight OR Obese Adults OR Adult with Obesity OR Adult Obesity OR Obesity in Adult OR Obese OR Adiposity) AND (S1 AND S2) - EBSCOhost Research Databases 223

SPORTDiscus with Full Text; MEDLINE with Full Text; Open Dissertations

S2 Obesity OR Overweight OR Excess Weight OR Obese Adults OR Adult with Obesity OR Adult Obesity OR Obesity in Adult OR Obese OR Adiposity EBSCOhost Research Databases 543,352

SPORTDiscus with Full Text; MEDLINE with Full Text; Open Dissertations

S1 Time Restricted Eating OR Eating, Time Restricted OR Time Restricted Fasting OR Fasting, Time Restricted

The effects of time-restricted eating on fat loss in adults with overweight and obese depend upon the eating window and intervention strategies: A systematic review and meta-analysis Yixun Xie

OR Restricted Fastings, Time OR Time Restricted Feeding OR Feeding, Time Restricted OR Time Restricted Feedings OR Time Restricted Meal OR Time Restricted Diet OR Early Time-Restricted Eating EBSCOhost Research Databases 636 SPORTDiscus with Full Text; MEDLINE with Full Text; Open Dissertations

Web of science

Entitlements # Search Query Database Results Date Run

- WOS: 1985 to 2024
- GRANTS: 1953 to 2024
- KJD: 1980 to 2024
- MEDLINE: 1950 to 2024
- PPRN: 1991 to 2024
- PQDT: 1637 to 2024
- SCIELO: 2002 to 2024" 1 "((((((((((TS=(Time Restricted Eating)) OR TS=(Eating, Time Restricted)) OR TS=(Time Restricted Fasting)) OR TS=(Fasting, Time Restricted)) OR TS=(Restricted Fastings, Time)) OR TS=(Time Restricted Feeding)) OR TS=(Feeding, Time Restricted)) OR TS=(Time Restricted Feedings)) OR TS=(Time Restricted Meal)) OR TS=(Time Restricted Diet)) OR TS=(Early Time-Restricted Eating) and Preprint Citation Index (Exclude – Database)" All Databases 22565

" 2 "((((((((TS=(Obesity)) OR TS=(Overweight)) OR TS=(Excess Weight)) OR TS=(Obese Adults)) OR TS=(Adult with Obesity)) OR TS=(Adult Obesity)) OR TS=(Obesity in Adult)) OR TS=(Obese)) OR TS=(Adiposity) and Preprint Citation Index (Exclude – Database)" All Databases 943463

" 3 "#2 AND #1 and Preprint Citation Index (Exclude – Database)" All Databases 4111

" 4 "#2 AND #1 and Preprint Citation Index (Exclude – Database)" All Databases 4111

" 5 "#2 AND #1 and Preprint Citation Index (Exclude – Database) and English (Languages)" All Databases 4029

" 6 "#2 AND #1 and Preprint Citation Index (Exclude – Database) and English (Languages) and Humans (MeSH Headings)" All Databases 1639

" 7 "#2 AND #1 and Preprint Citation Index (Exclude – Database) and English (Languages) and Humans (MeSH Headings) and Article or Other or Clinical Trial or Early Access (Document Types) " All Databases 1636

" 8 "#2 AND #1 and Preprint Citation Index (Exclude – Database)" All Databases 4111

" 9 "#2 AND #1 and Preprint Citation Index (Exclude – Database) and Article or Clinical Trial or Other (Document Types)" All Databases 3279

" 10 "#2 AND #1 and Preprint Citation Index (Exclude – Database) and Article or Clinical Trial or Other (Document Types) and Web of Science Core Collection (Database)" All Databases 3130

" 11 "#2 AND #1 and Preprint Citation Index (Exclude – Database) and Article or Clinical Trial or Other (Document Types) and Web of Science Core Collection (Database) and English (Languages) " All Databases 3102

" 12 "#2 AND #1 and Preprint Citation Index (Exclude – Database) and Article or Clinical Trial or Other (Document Types) and Web of Science Core Collection (Database) and English (Languages) and Humans (MeSH Headings) " All Databases 1575

" 13 "#2 AND #1 and Preprint Citation Index (Exclude – Database) and Article or Clinical Trial or Other (Document Types) and Web of Science Core Collection (Database) and English (Languages) and Humans (MeSH Headings) and 2014 or 2015 or 2016 or 2017 or 2018 or 2019 or 2020 or 2021 or 2022 or 2023 or 2024

The effects of time-restricted eating on fat loss in adults with overweight and obese depend upon the eating window and intervention strategies: A systematic review and meta-analysis Yixun Xie

(Publication Years)" All Databases 887
