# Supplementary material for: The Effects of Time-Restricted Eating on Fat Loss in Adults with Overweight and Obese Depend upon the Eating Window and Intervention Strategies: A Systematic Review and Meta-Analysis
Source: Nutrients. 2024 Oct 5;16(19):3390. doi: 10.3390/nu16193390 (PMC11478505; doi:10.3390/nu16193390)
Supplement: Supplementary file 1 [file nutrients-16-03390-s001.zip › Supplement S3.pdf]

The effects of time-restricted eating on fat loss in adults with overweight and obese depend upon the eating window and intervention strategies: A systematic review and meta-analysis Yixun Xie

Body Fat Percentage (BFP) Calculation

To calculate the Body Fat Percentage (BFP), use the following formula:

$$\mathrm{BFP} = \left(\frac{\text{Fat Mass}}{\text{Weight}}\right) \times 100\%$$

Error Calculation

To calculate the error in the Body Fat Percentage, use the error propagation formula:

$$\Delta\mathrm{BFP} = \mathrm{BFP} \times \sqrt{\left(\frac{\Delta\text{Fat Mass}}{\text{Fat Mass}}\right)^2 + \left(\frac{\Delta\text{Weight}}{\text{Weight}}\right)^2}$$
